# {μ-6,6′-Dimeth­oxy-2,2′-[ethane-1,2-diyl­bis(nitrilo­methanylyl­idene)]diphenolato-1κ^4^ *O* ^6^,*O* ^1^,*O* ^1′^,*O* ^6′^;2κ^4^ *O* ^1^,*N*,*N*′,*O* ^1′^}(methanol-1κ*O*)(tetra­fluoridoborato-1κ^2^ *F*,*F*′)-2-copper(II)-1-sodium

**DOI:** 10.1107/S160053681102842X

**Published:** 2011-07-23

**Authors:** Ting Gao, Yu Yang, Po Gao, Ju-Wen Zhang, Jing-Lin Yang

**Affiliations:** a School of Chemistry and Materials Science, Heilongjiang University, Harbin 150080, People’s Republic of China

## Abstract

In the dinuclear salen-type title complex, [CuNa(BF_4_)(C_18_H_18_N_2_O_4_)(CH_3_OH)], the Cu^II^ atom is chelated by two O atoms and two N atoms of the deprotonated Schiff base in a square-planar geometry. The Na atom is seven-coordinate as it is linked to four O atoms of the same Schiff base ligand, one O atom of the methanol and two tetra­fluorido­borate F atoms. The remaining two F atoms of the anion are disordered over two sites in a 0.598 (18):0.402 (18) ratio.

## Related literature

For similar copper–sodium complexes, see: Hazra *et al.* (2009 ▶); Sasmal *et al.* (2010 ▶).
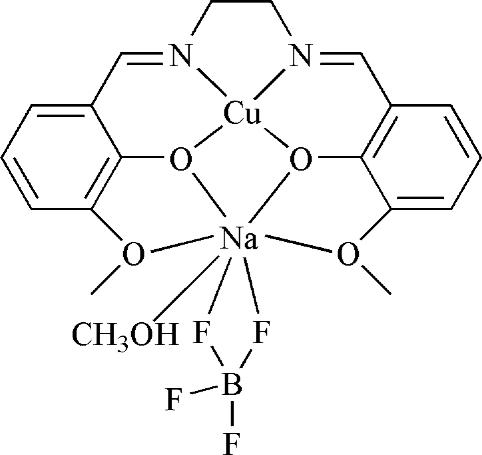


## Experimental

### 

#### Crystal data

[CuNa(BF_4_)(C_18_H_18_N_2_O_4_)(CH_4_O)]
*M* *_r_* = 531.73
Monoclinic, 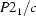

*a* = 12.885 (3) Å
*b* = 14.248 (5) Å
*c* = 13.508 (7) Å
β = 117.544 (17)°
*V* = 2198.7 (14) Å^3^
*Z* = 4
Mo *K*α radiation
μ = 1.08 mm^−1^
*T* = 293 K
0.33 × 0.27 × 0.25 mm

#### Data collection

Rigaku R-AXIS RAPID diffractometer
Absorption correction: multi-scan (*ABSCOR*; Higashi, 1995 ▶) *T*_min_ = 0.720, *T*_max_ = 0.778
20737 measured reflections
5016 independent reflections
3439 reflections with *I* > 2σ(*I*)
*R*_int_ = 0.057

#### Refinement

*R*[*F* ^2^ > 2σ(*F* ^2^)] = 0.050
*wR*(*F* ^2^) = 0.130
*S* = 1.05
5016 reflections
320 parameters
36 restraints
H-atom parameters constrained
Δρ_max_ = 0.49 e Å^−3^
Δρ_min_ = −0.77 e Å^−3^

### 

Data collection: *RAPID-AUTO* (Rigaku, 1998 ▶); cell refinement: *RAPID-AUTO*; data reduction: *CrystalClear* (Rigaku/MSC, 2002 ▶); program(s) used to solve structure: *SHELXS97* (Sheldrick, 2008 ▶); program(s) used to refine structure: *SHELXL97* (Sheldrick, 2008 ▶); molecular graphics: *SHELXTL* (Sheldrick, 2008 ▶); software used to prepare material for publication: *SHELXL97*.

## Supplementary Material

Crystal structure: contains datablock(s) global, I. DOI: 10.1107/S160053681102842X/ng5196sup1.cif

Structure factors: contains datablock(s) I. DOI: 10.1107/S160053681102842X/ng5196Isup2.hkl

Additional supplementary materials:  crystallographic information; 3D view; checkCIF report

## Figures and Tables

**Table 1:** Selected bond lengths (Å)

Cu1—O1	1.877 (2)
Cu1—O3	1.887 (2)
Cu1—N2	1.917 (3)
Cu1—N1	1.921 (3)
F1—Na1	2.533 (4)
F2—Na1	2.432 (3)
Na1—O1	2.319 (2)
Na1—O5	2.338 (3)
Na1—O3	2.349 (2)
Na1—O2	2.571 (3)
Na1—O4	2.662 (3)

**Fig. 1.**
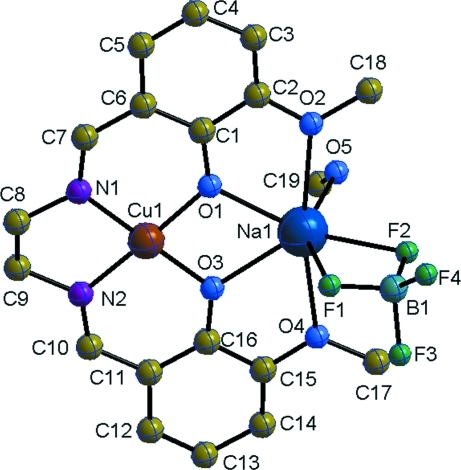
The molecular structure of the the title compound, showing 30% probability displacement ellipsoids.

## supplementary crystallographic information

### Comment

In continuation of our study of salen-type Cu—Na heterodinuclear complexes (Hazra *et al.*, 2009; Sasmal *et al.*, 2010), we present here the crystal structure of the title compound. As shown in Fig. 1, the hexadentate Schiff base ligand links Cu and Na atoms into a dinuclear complex through two phenolate O atoms. The Na^I^ centre in (I) is seven-coordinated by four O atoms from the ligand, one O atom from the methanol and two F atoms from the tetrafluoroborate, which is similar with the bonding reported for another copper-sodium complex of the similar ligand (Sasmal *et al.*, 2010). The Cu^II^ center is four-coordinate by two N atoms, two O atoms from the ligand giving rise to a square geometry.

### Experimental

The title complex was obtained by the treatment of copper(II) acetate monohydrate (0.050 g, 0.25 mmol) with the Schiff base (0.082 g, 0.25 mmol) in water/methanol (5:15). The first two reactants were stirred for 2 h, and the mixture was stirred for another 3 h after the addition of sodium (I) tetrafluoroborate (0.052 g, 0.25 mmol). The reaction mixture was filtered; diethyl ether was allowed to diffuse slowly into the solution of the filtrate. Single crystals were obtained after several days. Analysis calculated for for C19H22BCuF4N2NaO5 C_19_H_22_B_1Cu_1_F_1_N_2_Na_O_5_: C 42.92, H 4.17, N 5.27%; found: C 42.38, H 4.38, N 5.00%.

### Refinement

Two F atoms disordered in two positions with occupation of 0.6 to F3, F4 and 0.4 t0 F3' F4'. H atoms bound to C atoms were placed in calculated positions and treated as riding on their parent atoms, with C—H = 0.93 Å (aromatic C), C—H = 0.97 Å (methylene C), and with *U*_iso_(H) = 1.2Ueq(C) or C—H = 0.96 Å (methly C) and with *U*_iso_(H) = 1.5Ueq(C).

### Crystal data

**Table:**

[CuNa(BF_4_)(C_18_H_18_N_2_O_4_)(CH_4_O)]	*F*(000) = 1084
*M**_r_* = 531.73	*D*_x_ = 1.606 Mg m^−^^3^
Monoclinic, *P*2_1_/*c*	Mo *K*α radiation, λ = 0.71073 Å
Hall symbol: -P 2ybc	Cell parameters from 12791 reflections
*a* = 12.885 (3) Å	θ = 3.0–27.5°
*b* = 14.248 (5) Å	µ = 1.08 mm^−^^1^
*c* = 13.508 (7) Å	*T* = 293 K
β = 117.544 (17)°	Block, brown
*V* = 2198.7 (14) Å^3^	0.33 × 0.27 × 0.25 mm
*Z* = 4	

### Data collection

**Table:**

Rigaku R-AXIS RAPID diffractometer	5016 independent reflections
Radiation source: fine-focus sealed tube	3439 reflections with *I* > 2σ(*I*)
graphite	*R*_int_ = 0.057
ω scans	θ_max_ = 27.5°, θ_min_ = 3.0°
Absorption correction: multi-scan (*ABSCOR*; Higashi, 1995)	*h* = −16→16
*T*_min_ = 0.720, *T*_max_ = 0.778	*k* = −18→18
20737 measured reflections	*l* = −17→17

### Refinement

**Table:**

Refinement on *F*^2^	Primary atom site location: structure-invariant direct methods
Least-squares matrix: full	Secondary atom site location: difference Fourier map
*R*[*F*^2^ > 2σ(*F*^2^)] = 0.050	Hydrogen site location: inferred from neighbouring sites
*wR*(*F*^2^) = 0.130	H-atom parameters constrained
*S* = 1.05	*w* = 1/[σ^2^(*F*_o_^2^) + (0.0664*P*)^2^ + 0.3095*P*] where *P* = (*F*_o_^2^ + 2*F*_c_^2^)/3
5016 reflections	(Δ/σ)_max_ < 0.001
320 parameters	Δρ_max_ = 0.49 e Å^−^^3^
36 restraints	Δρ_min_ = −0.77 e Å^−^^3^

### Special details

**Table:**

Geometry. All e.s.d.'s (except the e.s.d. in the dihedral angle between two l.s. planes) are estimated using the full covariance matrix. The cell e.s.d.'s are taken into account individually in the estimation of e.s.d.'s in distances, angles and torsion angles; correlations between e.s.d.'s in cell parameters are only used when they are defined by crystal symmetry. An approximate (isotropic) treatment of cell e.s.d.'s is used for estimating e.s.d.'s involving l.s. planes.
Refinement. ISOR 0.005 F2 F3 F4 F3' F4' ISOR 0.001 F1SPLIT F3 F4 WITH THE OCCUPATION OF 0.60 FOR F3 AND F4, WHILE 0.40 FOR F3' AND F4'Refinement of *F*^2^ against ALL reflections. The weighted *R*-factor *wR* and goodness of fit *S* are based on *F*^2^, conventional *R*-factors *R* are based on *F*, with *F* set to zero for negative *F*^2^. The threshold expression of *F*^2^ > σ(*F*^2^) is used only for calculating *R*-factors(gt) *etc*. and is not relevant to the choice of reflections for refinement. *R*-factors based on *F*^2^ are statistically about twice as large as those based on *F*, and *R*- factors based on ALL data will be even larger.

### Fractional atomic coordinates and isotropic or equivalent isotropic displacement parameters (Å^2^)

**Table:**

	*x*	*y*	*z*	*U*_iso_*/*U*_eq_	Occ. (<1)
B1	0.4588 (4)	0.6368 (4)	0.7395 (4)	0.0644 (12)	
C1	0.1383 (3)	0.8585 (2)	0.4357 (3)	0.0394 (7)	
C2	0.2498 (3)	0.8999 (2)	0.4724 (3)	0.0428 (7)	
C3	0.2622 (3)	0.9962 (2)	0.4754 (3)	0.0520 (9)	
H3	0.3361	1.0228	0.5014	0.062*	
C4	0.1641 (4)	1.0532 (2)	0.4394 (3)	0.0603 (10)	
H4	0.1729	1.1180	0.4417	0.072*	
C5	0.0555 (4)	1.0158 (2)	0.4011 (3)	0.0535 (9)	
H5	−0.0093	1.0552	0.3758	0.064*	
C6	0.0398 (3)	0.9171 (2)	0.3992 (3)	0.0429 (7)	
C7	−0.0766 (3)	0.8817 (2)	0.3595 (3)	0.0478 (8)	
H7	−0.1368	0.9255	0.3371	0.057*	
C8	−0.2263 (3)	0.7663 (3)	0.3176 (4)	0.0614 (10)	
H8A	−0.2794	0.8099	0.2621	0.074*	
H8B	−0.2418	0.7675	0.3814	0.074*	
C9	−0.2462 (3)	0.6683 (3)	0.2691 (4)	0.0608 (10)	
H9A	−0.3074	0.6376	0.2800	0.073*	
H9B	−0.2713	0.6717	0.1895	0.073*	
C10	−0.1397 (3)	0.5225 (2)	0.3216 (3)	0.0466 (8)	
H10	−0.2126	0.4936	0.2929	0.056*	
C11	−0.0396 (3)	0.4631 (2)	0.3596 (3)	0.0415 (7)	
C12	−0.0573 (3)	0.3646 (2)	0.3493 (3)	0.0534 (9)	
H12	−0.1327	0.3409	0.3233	0.064*	
C13	0.0325 (4)	0.3045 (2)	0.3764 (3)	0.0603 (10)	
H13	0.0183	0.2403	0.3676	0.072*	
C14	0.1472 (4)	0.3382 (2)	0.4178 (3)	0.0535 (9)	
H14	0.2090	0.2966	0.4360	0.064*	
C15	0.1678 (3)	0.4333 (2)	0.4312 (3)	0.0430 (7)	
C16	0.0759 (3)	0.4983 (2)	0.4018 (3)	0.0395 (7)	
C17	0.3769 (3)	0.4155 (3)	0.5113 (4)	0.0727 (12)	
H17A	0.3755	0.3705	0.5636	0.109*	
H17B	0.4464	0.4530	0.5469	0.109*	
H17C	0.3763	0.3833	0.4486	0.109*	
C18	0.4534 (3)	0.8701 (3)	0.5375 (4)	0.0744 (13)	
H18A	0.4532	0.9091	0.4794	0.112*	
H18B	0.5053	0.8180	0.5503	0.112*	
H18C	0.4793	0.9061	0.6048	0.112*	
C19	0.3012 (5)	0.6111 (4)	0.2577 (4)	0.111 (2)	
H19A	0.2722	0.5537	0.2728	0.166*	
H19B	0.3612	0.5971	0.2366	0.166*	
H19C	0.2384	0.6438	0.1978	0.166*	
Cu1	0.00033 (3)	0.68991 (2)	0.38549 (3)	0.03933 (15)	
F1	0.3435 (3)	0.6599 (2)	0.6971 (3)	0.1045 (9)	
F2	0.4815 (2)	0.6418 (2)	0.6487 (2)	0.0929 (8)	
F3	0.4532 (9)	0.5441 (4)	0.7756 (5)	0.102 (3)	0.598 (18)
F4	0.5310 (7)	0.6847 (8)	0.8305 (7)	0.099 (2)	0.598 (18)
F3'	0.5214 (16)	0.5676 (9)	0.7911 (9)	0.124 (5)	0.402 (18)
F4'	0.5110 (9)	0.7239 (8)	0.8004 (10)	0.081 (3)	0.402 (18)
N1	−0.1050 (2)	0.79428 (19)	0.3521 (2)	0.0458 (7)	
N2	−0.1379 (2)	0.61370 (19)	0.3237 (2)	0.0448 (6)	
Na1	0.28783 (11)	0.66051 (9)	0.49108 (11)	0.0476 (3)	
O1	0.13430 (18)	0.76601 (14)	0.4389 (2)	0.0481 (6)	
O2	0.3381 (2)	0.83617 (16)	0.5045 (2)	0.0547 (7)	
O3	0.10354 (18)	0.58764 (14)	0.4150 (2)	0.0457 (6)	
O4	0.27683 (19)	0.47424 (15)	0.4739 (2)	0.0546 (6)	
O5	0.3468 (3)	0.6662 (2)	0.3515 (2)	0.0732 (8)	
H1	0.3777	0.7166	0.3444	0.110*	

### Atomic displacement parameters (Å^2^)

**Table:**

	*U*^11^	*U*^22^	*U*^33^	*U*^12^	*U*^13^	*U*^23^
B1	0.048 (2)	0.073 (3)	0.063 (3)	0.015 (2)	0.018 (2)	0.006 (3)
C1	0.0422 (16)	0.0348 (15)	0.0385 (17)	0.0012 (13)	0.0165 (14)	0.0022 (13)
C2	0.0480 (17)	0.0348 (15)	0.0422 (17)	−0.0006 (14)	0.0178 (14)	0.0026 (14)
C3	0.063 (2)	0.0373 (16)	0.059 (2)	−0.0128 (16)	0.0319 (18)	−0.0068 (16)
C4	0.090 (3)	0.0321 (16)	0.076 (3)	0.0003 (18)	0.053 (2)	−0.0002 (17)
C5	0.073 (2)	0.0345 (16)	0.065 (2)	0.0152 (17)	0.043 (2)	0.0065 (16)
C6	0.0543 (18)	0.0368 (15)	0.0432 (18)	0.0078 (14)	0.0272 (15)	0.0050 (14)
C7	0.0456 (17)	0.0494 (19)	0.051 (2)	0.0160 (15)	0.0244 (16)	0.0147 (16)
C8	0.0354 (17)	0.070 (2)	0.077 (3)	0.0132 (17)	0.0247 (18)	0.009 (2)
C9	0.0308 (16)	0.073 (2)	0.067 (2)	−0.0030 (16)	0.0126 (16)	0.001 (2)
C10	0.0397 (16)	0.055 (2)	0.0420 (18)	−0.0143 (15)	0.0164 (14)	−0.0059 (15)
C11	0.0465 (16)	0.0403 (16)	0.0398 (17)	−0.0078 (14)	0.0218 (14)	−0.0048 (14)
C12	0.065 (2)	0.0458 (19)	0.055 (2)	−0.0210 (17)	0.0327 (19)	−0.0045 (16)
C13	0.084 (3)	0.0321 (16)	0.070 (3)	−0.0097 (18)	0.040 (2)	−0.0048 (17)
C14	0.076 (3)	0.0350 (15)	0.059 (2)	0.0044 (16)	0.038 (2)	0.0039 (16)
C15	0.0510 (17)	0.0369 (15)	0.0497 (19)	−0.0013 (14)	0.0306 (16)	0.0006 (14)
C16	0.0462 (17)	0.0378 (15)	0.0371 (16)	−0.0022 (13)	0.0216 (14)	−0.0015 (14)
C17	0.054 (2)	0.062 (2)	0.108 (4)	0.0147 (19)	0.043 (2)	0.013 (2)
C18	0.044 (2)	0.067 (2)	0.101 (3)	−0.0151 (19)	0.024 (2)	−0.015 (2)
C19	0.142 (5)	0.108 (4)	0.074 (3)	−0.034 (4)	0.043 (3)	−0.018 (3)
Cu1	0.02938 (19)	0.0377 (2)	0.0470 (2)	0.00197 (15)	0.01437 (17)	0.00150 (17)
F1	0.0963 (12)	0.1098 (12)	0.1087 (13)	0.0003 (9)	0.0486 (9)	0.0025 (9)
F2	0.0854 (17)	0.125 (2)	0.0776 (16)	0.0071 (16)	0.0454 (14)	−0.0047 (16)
F3	0.116 (5)	0.078 (3)	0.098 (4)	0.007 (3)	0.039 (3)	0.036 (2)
F4	0.093 (4)	0.110 (5)	0.080 (3)	−0.022 (3)	0.028 (3)	−0.007 (3)
F3'	0.124 (6)	0.108 (6)	0.120 (5)	0.018 (4)	0.040 (4)	0.025 (4)
F4'	0.078 (4)	0.075 (5)	0.081 (5)	−0.004 (3)	0.028 (3)	−0.023 (4)
N1	0.0359 (13)	0.0518 (16)	0.0495 (16)	0.0087 (12)	0.0195 (12)	0.0092 (13)
N2	0.0328 (13)	0.0533 (16)	0.0444 (15)	−0.0012 (12)	0.0147 (12)	−0.0007 (13)
Na1	0.0361 (6)	0.0456 (7)	0.0581 (8)	0.0021 (5)	0.0192 (6)	0.0034 (6)
O1	0.0350 (11)	0.0301 (10)	0.0698 (15)	0.0034 (9)	0.0163 (11)	0.0073 (11)
O2	0.0383 (12)	0.0416 (12)	0.0728 (17)	−0.0061 (10)	0.0162 (12)	−0.0005 (12)
O3	0.0336 (10)	0.0344 (10)	0.0653 (15)	−0.0019 (9)	0.0195 (10)	−0.0025 (10)
O4	0.0418 (12)	0.0421 (12)	0.0812 (18)	0.0065 (10)	0.0294 (12)	0.0047 (12)
O5	0.094 (2)	0.0694 (16)	0.0692 (19)	−0.0225 (16)	0.0490 (17)	−0.0134 (15)

### Geometric parameters (Å, °)

**Table:**

B1—F3'	1.260 (10)	C12—H12	0.9300
B1—F4	1.336 (8)	C13—C14	1.402 (6)
B1—F1	1.363 (5)	C13—H13	0.9300
B1—F2	1.387 (6)	C14—C15	1.376 (4)
B1—F3	1.422 (8)	C14—H14	0.9300
B1—F4'	1.469 (10)	C15—O4	1.377 (4)
B1—Na1	3.064 (5)	C15—C16	1.409 (4)
C1—O1	1.320 (4)	C16—O3	1.311 (4)
C1—C6	1.404 (4)	C17—O4	1.419 (4)
C1—C2	1.414 (4)	C17—H17A	0.9600
C2—O2	1.360 (4)	C17—H17B	0.9600
C2—C3	1.380 (4)	C17—H17C	0.9600
C3—C4	1.388 (5)	C18—O2	1.424 (4)
C3—H3	0.9300	C18—H18A	0.9600
C4—C5	1.356 (5)	C18—H18B	0.9600
C4—H4	0.9300	C18—H18C	0.9600
C5—C6	1.420 (4)	C19—O5	1.371 (6)
C5—H5	0.9300	C19—H19A	0.9600
C6—C7	1.430 (5)	C19—H19B	0.9600
C7—N1	1.289 (4)	C19—H19C	0.9600
C7—H7	0.9300	Cu1—O1	1.877 (2)
C8—N1	1.465 (4)	Cu1—O3	1.887 (2)
C8—C9	1.513 (5)	Cu1—N2	1.917 (3)
C8—H8A	0.9700	Cu1—N1	1.921 (3)
C8—H8B	0.9700	Cu1—Na1	3.3236 (15)
C9—N2	1.464 (4)	F1—Na1	2.533 (4)
C9—H9A	0.9700	F2—Na1	2.432 (3)
C9—H9B	0.9700	Na1—O1	2.319 (2)
C10—N2	1.299 (4)	Na1—O5	2.338 (3)
C10—C11	1.426 (5)	Na1—O3	2.349 (2)
C10—H10	0.9300	Na1—O2	2.571 (3)
C11—C16	1.417 (4)	Na1—O4	2.662 (3)
C11—C12	1.418 (4)	O5—H1	0.8472
C12—C13	1.347 (5)		
			
F3'—B1—F4	84.0 (6)	O2—C18—H18A	109.5
F3'—B1—F1	135.7 (11)	O2—C18—H18B	109.5
F4—B1—F1	115.0 (6)	H18A—C18—H18B	109.5
F3'—B1—F2	100.8 (8)	O2—C18—H18C	109.5
F4—B1—F2	116.5 (6)	H18A—C18—H18C	109.5
F1—B1—F2	104.5 (4)	H18B—C18—H18C	109.5
F3'—B1—F3	37.3 (7)	O5—C19—H19A	109.5
F4—B1—F3	106.4 (6)	O5—C19—H19B	109.5
F1—B1—F3	98.6 (6)	H19A—C19—H19B	109.5
F2—B1—F3	114.6 (5)	O5—C19—H19C	109.5
F3'—B1—F4'	109.9 (7)	H19A—C19—H19C	109.5
F4—B1—F4'	27.1 (4)	H19B—C19—H19C	109.5
F1—B1—F4'	99.1 (5)	O1—Cu1—O3	86.09 (9)
F2—B1—F4'	102.9 (7)	O1—Cu1—N2	177.21 (11)
F3—B1—F4'	132.5 (7)	O3—Cu1—N2	94.40 (11)
F3'—B1—Na1	130.1 (7)	O1—Cu1—N1	93.86 (11)
F4—B1—Na1	142.5 (6)	O3—Cu1—N1	178.71 (12)
F1—B1—Na1	54.7 (2)	N2—Cu1—N1	85.59 (12)
F2—B1—Na1	50.6 (2)	O1—Cu1—Na1	42.57 (7)
F3—B1—Na1	110.7 (4)	O3—Cu1—Na1	43.55 (6)
F4'—B1—Na1	115.5 (6)	N2—Cu1—Na1	137.94 (9)
O1—C1—C6	124.2 (3)	N1—Cu1—Na1	136.43 (9)
O1—C1—C2	117.0 (3)	B1—F1—Na1	99.3 (3)
C6—C1—C2	118.8 (3)	B1—F2—Na1	103.3 (2)
O2—C2—C3	125.8 (3)	C7—N1—C8	120.7 (3)
O2—C2—C1	113.5 (3)	C7—N1—Cu1	125.8 (2)
C3—C2—C1	120.7 (3)	C8—N1—Cu1	113.4 (2)
C2—C3—C4	119.8 (3)	C10—N2—C9	121.1 (3)
C2—C3—H3	120.1	C10—N2—Cu1	125.4 (2)
C4—C3—H3	120.1	C9—N2—Cu1	113.3 (2)
C5—C4—C3	121.1 (3)	O1—Na1—O5	106.39 (11)
C5—C4—H4	119.5	O1—Na1—O3	66.79 (8)
C3—C4—H4	119.5	O5—Na1—O3	107.61 (11)
C4—C5—C6	120.6 (3)	O1—Na1—F2	136.25 (11)
C4—C5—H5	119.7	O5—Na1—F2	97.21 (12)
C6—C5—H5	119.7	O3—Na1—F2	139.01 (11)
C1—C6—C5	119.0 (3)	O1—Na1—F1	96.01 (11)
C1—C6—C7	122.8 (3)	O5—Na1—F1	148.64 (12)
C5—C6—C7	118.1 (3)	O3—Na1—F1	101.22 (11)
N1—C7—C6	125.5 (3)	F2—Na1—F1	51.90 (11)
N1—C7—H7	117.3	O1—Na1—O2	62.75 (8)
C6—C7—H7	117.3	O5—Na1—O2	81.83 (10)
N1—C8—C9	109.3 (3)	O3—Na1—O2	129.21 (9)
N1—C8—H8A	109.8	F2—Na1—O2	85.51 (10)
C9—C8—H8A	109.8	F1—Na1—O2	89.52 (10)
N1—C8—H8B	109.8	O1—Na1—O4	128.03 (9)
C9—C8—H8B	109.8	O5—Na1—O4	89.32 (10)
H8A—C8—H8B	108.3	O3—Na1—O4	61.24 (7)
N2—C9—C8	110.0 (3)	F2—Na1—O4	87.64 (9)
N2—C9—H9A	109.7	F1—Na1—O4	93.96 (10)
C8—C9—H9A	109.7	O2—Na1—O4	168.04 (10)
N2—C9—H9B	109.7	O1—Na1—B1	118.99 (12)
C8—C9—H9B	109.7	O5—Na1—B1	123.34 (14)
H9A—C9—H9B	108.2	O3—Na1—B1	119.99 (13)
N2—C10—C11	125.6 (3)	F2—Na1—B1	26.14 (12)
N2—C10—H10	117.2	F1—Na1—B1	26.03 (12)
C11—C10—H10	117.2	O2—Na1—B1	90.00 (12)
C16—C11—C12	118.8 (3)	O4—Na1—B1	88.16 (12)
C16—C11—C10	122.8 (3)	O1—Na1—Cu1	33.19 (5)
C12—C11—C10	118.3 (3)	O5—Na1—Cu1	111.29 (10)
C13—C12—C11	121.5 (3)	O3—Na1—Cu1	33.61 (5)
C13—C12—H12	119.2	F2—Na1—Cu1	151.40 (9)
C11—C12—H12	119.2	F1—Na1—Cu1	99.51 (9)
C12—C13—C14	120.4 (3)	O2—Na1—Cu1	95.86 (6)
C12—C13—H13	119.8	O4—Na1—Cu1	94.84 (6)
C14—C13—H13	119.8	B1—Na1—Cu1	125.32 (11)
C15—C14—C13	119.6 (3)	C1—O1—Cu1	127.34 (19)
C15—C14—H14	120.2	C1—O1—Na1	128.05 (19)
C13—C14—H14	120.2	Cu1—O1—Na1	104.24 (10)
C14—C15—O4	124.7 (3)	C2—O2—C18	118.1 (3)
C14—C15—C16	121.6 (3)	C2—O2—Na1	118.72 (18)
O4—C15—C16	113.6 (3)	C18—O2—Na1	123.0 (2)
O3—C16—C15	117.4 (3)	C16—O3—Cu1	126.9 (2)
O3—C16—C11	124.6 (3)	C16—O3—Na1	130.14 (19)
C15—C16—C11	118.0 (3)	Cu1—O3—Na1	102.84 (9)
O4—C17—H17A	109.5	C15—O4—C17	118.8 (3)
O4—C17—H17B	109.5	C15—O4—Na1	117.52 (18)
H17A—C17—H17B	109.5	C17—O4—Na1	123.6 (2)
O4—C17—H17C	109.5	C19—O5—Na1	124.5 (3)
H17A—C17—H17C	109.5	C19—O5—H1	113.8
H17B—C17—H17C	109.5	Na1—O5—H1	117.7
